# Supplementary material for: Screening of Native Trichoderma Species for Nickel and Copper Bioremediation Potential Determined by FTIR and XRF
Source: Microorganisms. 2023 Mar 22;11(3):815. doi: 10.3390/microorganisms11030815 (PMC10053837; doi:10.3390/microorganisms11030815)
Supplement: Supplementary file 1 [file microorganisms-11-00815-s001.zip › microorganisms-2187757-supplementary.pdf]

Supplementary Materials

Table S1. Morphological characteristics of *Trichoderma* spp. under different concentrations Cu and Ni.

	Treatments	Phialide length (μm)		Phialide width (μm)		Conidia length (μm)	Conidia width (μm)
		Apical phialide	Lateral phialide	Apical phialide	Lateral phialide		
20696 Trichoderma harzianum	Control	8.98 ± 2.00 a	6.72 ± 0.73 a	2.49 ± 0.04 a	2.77 ± 0.22 a	2.87 ± 0.30 b	2.47 ± 0.39 a
	60 mg/L Cu	11.21 ± 3.46 a	6.70 ± 1.17 a	2.99 ± 0.42 a	3.13 ± 0.49 a	3.01 ± 0.13 b	2.78 ± 0.08 a
	120 mg/L Cu	9.94 ± 1.58 a	5.54 ± 0.71 a	2.15 ± 0.11 a	2.89 ± 032 a	2.87 ± 0.25 b	2.38 ± 0.19 a
20660 Trichoderma harzianum	Control	10.12 ± 2.77 a	5.57 ± 0.85 a	2.37 ± 0.38 a	2.98 ± 0.38 a	2.72 ± 0.30 b	2.31 ± 0.23 a
	60 mg/L Cu	11.28 ± 2.54 a	6.39 ± 0.59 a	2.29 ± 0.33 a	3.00 ± 0.28 a	2.93 ± 0.35 b	2.48 ± 0.22 a
	120 mg/L Cu	10.03 ± 3.16 a	6.26 ± 0.67 a	2.22 ± 0.21 a	2.84 ± 0.21 a	3.00 ± 0.31 b	2.42 ± 0.19 a
20664 Trichoderma longibrachiatum	Control	7.83 ± 1.48 a		2.53 ± 0.21 a		3.90 ± 0.16 a	2.48 ± 0.24 a
	60 mg/L Ni	8.06 ± 0.54 a		2.81 ± 0.37 a		3.94 ± 0.47 a	2.45 ± 0.15 a
	120 mg/L Ni	7.87 ± 1.57 a		2.61 ± 0.16 a		3.85 ± 0.33 a	2.49 ± 0.21 a
22669 Trichoderma longibrachiatum	Control	8.11 ± 1.70 a		2.64 ± 0.25 a		4.14 ± 0.42 a	2.39 ± 0.25 a
	60 mg/L Ni	7.91 ± 1.57 a		2.71 ± 0.23 a		4.26 ± 0.50 a	2.36 ± 0.14 a
	120 mg/L Ni	8.28 ± 0.96 a		2.66 ± 0.18 a		4.29 ± 0.56 a	2.48 ± 0.18 a
22665 Trichoderma longibrachiatum	Control	6.18 ± 1.02 a		2.34 ± 0.33 a		3.35 ± 0.31 ab	2.54 ± 0.27 a
	1mmol Ni	6.46 ± 1.40 a		2.73 ± 0.39 a		3.22 ± 0.31 ab	2.66 ± 0.37 a
	2mmol Ni	5.65 ± 2.31 a		2.78 ± 0.26 a		3.20 ± 0.23 ab	2.68 ± 0.36 a

The statistical data were analyzed with the use of the one-way ANOVA and Tukey's post hoc tests and a statistically significant difference (p-value < 0.05) is denoted by a different letter in the table.
